# Novel Cucurbitane Triterpenes from the Tubers of *Hemsleya amabilis* with Their Cytotoxic Acitivity

**DOI:** 10.3390/molecules24020331

**Published:** 2019-01-17

**Authors:** Wei Feng, Yuan Zhou, Ling-Yu Zhou, Li-Ying Kang, Xiang Wang, Bao-Lin Li, Qing Li, Li-Ying Niu

**Affiliations:** School of Pharmaceutical Sciences, Hebei TCM Formula Granule Technology Innovation Center& TCM Formula Granule Research Center of Hebei Province University, Hebei University of Chinese Medicine, Shijiazhuang 050091, China; weifeng@hebcm.edu.cn (W.F.); 18832160770@163.com (Y.Z.); zhuangxzz@163.com (L.-Y.Z.); 13171895576@126.com (L.-Y.K.); wangxiangdea@126.com (X.W.); libaol2016@163.com (B.-L.L.); lqlqlqlqlq5@126.com (Q.L.)

**Keywords:** *Hemsleya amabilis*, cucurbitane-type, triterpenes, cytotoxic activity

## Abstract

Chemical research of the medicinal plant *Hemsleya amabilis* (Cucurbitaceae) yielded five new cucurbitane-type triterpenes hemslelis A–E (**1**–**5**) by silica gel column, ODS column, and semi-HPLC techniques. Their structures were determined by spectroscopic analysis and examined alongside existing data from prior studies. Compounds **1**–**5** were evaluated for their cytotoxic activities against three human tumor cell lines, Hela, HCT-8, and HepG-2, with the IC_50_ ranging from 5.9 to 33.9 μM compared to Cisplatin.

## 1. Introduction

*Hemsleya*, a genus of Cucurbitaceae family, is comprised of more than thirty species in tropical and subtropical regions of China [1]. Most tubers of the plants in this genus have been used as traditional medicine in ethnic minority areas of China. Previous phytochemical evaluations on this genus have disclosed the presence of abundant compounds, such as diterpenes, oleanane, and cucurbitane-type triterpenes [2,3,4]. Among these components, cucurbitane triterpenes have shown potent cytotoxic activity. Hemslecin A (also called cucurbitacin IIa) was reported to suppress cancer cell growth in vitro and reduce tumor size on mouse H22 liver cancer [5]. Some evaluations have indicated that the mechanisms of cucurbitacins’ activities includes the disruption of the Jaks-Stat (Janus kinase-signal transducer and activator of transcription) signaling pathway, and especially the STAT3 signaling pathway [6,7,8,9,10].

**Figure 1:**
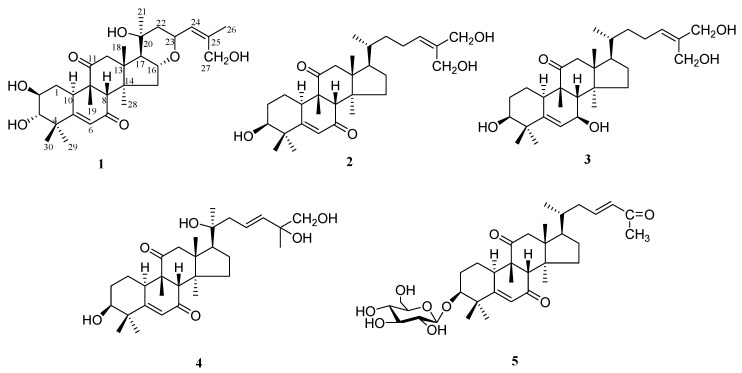
Structures of compounds **1**–**5**.

*Hemsleya amabilis*, a species of the genus *Hemsleya*, commonly known as “xue dan” in Yunnan province of China, has been long used as a part of “Dai” medicine and dispensed for bacillary dysentery, tuberculosis, stomachache, whooping cough, and bile duct infection (The Pharmacopoeia Commission of PRC, 2005). For the purpose of finding new bioactive cucurbitane triterpenes from this medicinal plant, we examined the ethanol extract of *H. amabilis* and isolated five new cucurbitane triterpenes, hemslelis A-E (**1–5**) (Figure 1). In this paper, we reported the isolation and structure elucidation of the new compounds as well as their cytotoxic activity.

## 2. Results

### 2.1. Structure Elucidation

**Figure 2:**
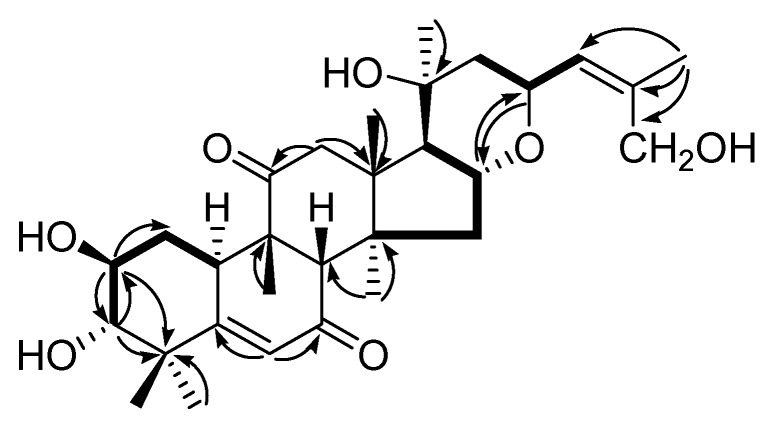
Key ^1^H-^1^H COSY (bold items) and HMBC (arrows) correlations of compound **1**.

Compound **1** was obtained as an amorphous solid with ${\left[\alpha \right]}_{D}^{20}$ + 73.1 (*c* = 0.1, MeOH). HRESIMS gave a sodium adduct ion peak at *m*/*z* 539.2942 [M + Na]^+^ (calcd. 539.2985 for C_30_H_44_O_7_Na) in the positive-ion mode, in conjunction with NMR data supported the molecular formula of C_30_H_44_O_7_. The IR spectrum displayed the functional groups of hydroxyl at 3447 cm^−1^, carbonyl at 1687 cm^−1^, and methyl at 2938 and 2854 cm^−1^. The ^1^H-NMR spectrum (Table 1, Supporting Information Figure S1) of **1** exhibited seven angular methyl signals at δ_H_ 1.23 (3H, s), 1.26 (3H, s), 1.36 (3H, s), 1.41 (3H, s), 1.45 (3H, s), 1.50 (3H, s), and 1.98 (3H, s), two olefinic protons at δ_H_ 6.43 (1H, s), 6.65 (1H, d, *J* = 6.6 Hz), and a set of oxygenated proton signals at δ_H_ 3.52 (1H, d, *J* = 8.4 Hz), 4.14 (1H, td, *J* = 1.2, 8.4 Hz), 5.17 (1H, m), 5.10 (1H, m), 4.50 (1H, d, *J* = 12.6 Hz), 4.57 (1H, d, *J* = 12.6 Hz). The ^13^C APT NMR spectrum (Table 1) displayed 30 signals including seven sp3 carbons, five sp2 carbons, nine sp carbons, and nine tetrasubstituted carbons (two carbonyl carbons at δ_C_ 200.3, 211.5; two olefinic carbons at δ_C_ 168.3, 139.3). All proton signals were assigned to the corresponding carbons through direct ^1^H and ^13^C correlations in the HSQC spectrum. The comparision of the above data with reported ones suggested that compound **1** was a polyhydroxy substituted △^5(6),24(25)^ cucurbitane triterpenoid [11,12,13,14]. Examinations of its ^1^H-^1^H COSY spectrum advanced the establishment of three fragments C-10-C-1-C-2-C-3, C-15-C-16-C-17, and C-22-C-23-C-24 (Figure 2), which further confirmed the basic skeleton of cucurbitane-type triterpenoid. In the HMBC spectrum, the correlations from δ_H_ 4.14 (1H, td, *J* = 1.2, 8.4 Hz, H-2) to C-1 (δ_C_ 34.2), C-3 (δ_C_ 80.8), and C-4 (δ_C_ 44.8), δ_H_ 3.52 (1H, d, *J* = 8.4 Hz, H-3) to C-2 (δ_C_ 70.5) and C-4 (δ_C_ 44.8) suggested the presence of hydroxyl groups at C-1 and C-2, respectively. Furthermore, HMBC correlations of H-6 with C-5 (δ_C_ 168.3) and C-7 (δ_C_ 200.3), H_3_-26 with C-24 (δ_C_ 129.0), C-25 (δ_C_ 139.3), and C-27 (δ_C_ 61.2) implied an α, β-unsaturated carbonyl moiety at C-5/6/7, double bond at C-24/25, and hydroxymethyl at C-27. Long-range correlations between δ_H_ 3.25 (1H, m, H-12a) and C-11 (δ_C_ 211.5) in the HMBC spectrum indicated that the hydroxy group at C-11 in the reported ones has been replaced by a carbonyl group in compound **1** [15,16]. C-15 was linked with C-23 through an O atom on the basis of the correlations from the proton signals at δ_H_ 5.17 (1H, m, H-16) to C-23 (δ_C_ 70.8) and δ_H_ 5.10 (1H, m, H-23) to C-16 (δ_C_ 70.9). Taking into consideration of cucurbitacins’ biogenesis, the stereochemistry of the tetracyclic system of **1** was established as shown and further confirmed by 2D NOESY experiment. The NOE correlations of H-2 with H-10, H-3 with H-1b, and H-1b with H_3_-19 indicated the β-orientation of OH-2 and the α-orientation of OH-3. The large coupling constants of H-2 and H-3 (*J* = 8.4 Hz) also supported the antiperiplanar relationship between them. The NOE enhancement of H-16 with H_3_-18 elucidated the configuration of oxygen bridge between C-16 and C-23. As a result, the structure of compound **1** was depicted as 2β, 3α, 20, 27-tetrahydroxycucurbita-16, 23-anhydrocucurbita-5, 24-diene-7, 11-dione, and named as hemslelis A.

Compound **2** was obtained as a white amorphous powder. The molecular formula C_30_H_46_O_5_ was analysed on the basis of its quasi-molecular ion [M + Na]^+^ at *m*/*z*: 509.3237 in the HRESIMS. Its ^1^H-NMR spectrum displayed six angular methyl protons at δ_H_ 0.70 (3H, s), 0.83 (3H, d, *J* = 6.6 Hz), 1.10 (3H, s), 1.21 (3H, s), 1.29 (3H, s), and 1.44 (3H, s), two olefinic protons at δ_H_ 6.47 (1H, s) and 5.90 (1H, t, *J* = 7.8 Hz). ^13^C APT displayed 30 carbons including six sp3 carbons, nine sp2 carbons, seven sp carbons, and eight quaternary carbons. All the data above were very similar to those of **1**, except for the disappearance of four oxygenated carbons (δ_C_ 70.5, 70.8, 70.9, and 72.7) and the appearance of one additional hydroxymethyl group singal (δ_C_ 65.7) in compound **2**. In the HMBC spectrum, the correlations from δ_H_ 3.77 (1H, m, H-3) to δ_C_ 29.6 (C-2) and 43.9 (C-4), δ_H_ 0.83 (3H, d, *J* = 6.6 Hz, H_3_-21) to δ_C_ 36.3 (C-20), and δ_H_ 5.90 (1H, t, *J* = 7.8 Hz, H-24) to δ_C_ 24.8 (C-23), 65.7 (C-25), and 58.8 (C-26), as well as the molecular formula C_30_H_46_O_5_, indicated the missing hydroxyl groups at C-2 and C-20, oxygen bridge at C-16 and C-23, and the extra hydroxymethyl group at C-24 in **2**. Taken together with the NOESY spectrum, the structure of **2** was established as 3β, 26, 27-trihydroxycucurbita-5, 24-dien-7,11-dione, and given the trivial name hemslelis B.

Compound **3** was obtained as an amorphous white powder, and its molecular formula was established as C_30_H_48_O_5_ on the basis of the positive molecular ion peak at *m*/*z* 511.3328 [M + Na]^+^ in the HRESMS. Its ^1^H- and ^13^C-NMR data (Table 1) were close to those of **2**, with the exception of the lack of one carbonyl ketone bond and the emergence of one oxygenated methine signal. In comparison with **2**, the signal for C-7 revealed a powerful upfield shift to δ_C_ 66.5 (−133.1 ppm), which indicated that the ketone group at C-7 in **2** was reduced to hydroxyl group in **3**. Taken together with ^1^H-^1^H COSY, HSQC, HMBC, and NOE spectra, the structure of compound **3** was determined to be 3β, 7β, 26, 27-tetrahydroxycucurbita-5, 24-dien-11-one, and named hemslelis C.

Compound **4** possesses the elemental composition C_30_H_46_O_6_, as established by HRESIMS and NMR examinations. Its ^1^H- and ^13^C-NMR (Table 1) data are close to those of compound **3**, with the exception of the double bond at C-24/25 and hydroxyl group at C-27 in **3**, which were moved to C-23/24 and C-25, respectively, in **4**, and the additional hydroxyl group at C-20 in **4**. Moreover, in the HMBC spectrum, the connections between H-26 (δ_H_ 3.93) and H_3_-27 (δ_H_ 1.64) to C-25 (δ_C_ 73.7) and C-24 (δ_C_ 139.9), as well as H_3_-21 (δ_H_ 1.46) to C-20 (δ_C_ 74.5), additionally verified the dissimilarity. The form of **4** was verified by examinations of its ^1^H-^1^H COSY, HSQC, HMBC, and NOE spectra and established as 3β, 20, 25, 26-tetrahydroxycucurbita-5, 23-dien-7, 11-dione, and named hemslelis D.

Compound **5** was isolated as a white amorphous powder. Its molecular formula was established as C_35_H_52_O_9_ by HRESIMS (observed *m*/*z* 639.3522 [M + Na]^+^, calcd. for 639.3509), requiring ten degrees of unsaturation. The NMR data of **5** were similar to those of compound **4**, except for the missing carbon of C-26 and hydroxyl group at C-20 in **5** which were confirmed by the ^13^C-NMR data (δ_C_ 36.4, C-20) and HMBC correlations from δ_H_ 2.26 (H-27) to δ_C_ 198.2 (C-25). The sugar moiety was located at C-3 on the basis of the correlation between the proton signal at δ_H_ 3.71 (H-3) and anomeric carbon at δ_C_ 107.6. The type and absolute configuration of the sugar was identified as d-glucose on the basis of the TLC method comparison with authentic monosaccharides (CHCl_3_:MeOH:H_2_O = 3:2:0.2, visualization with ethanol-5% H_2_SO_4_ spraying), followed by gas chromatography. The NOESY correlations of H-3/Me-29 and Me-29/H-10 confirmed that H-3 was α-orientation. Therefore, compound **5** was determined to be 3-*O*-β-d-glucopyranoside-cucurbita-5, 23-diene-7, 11, 25-trione, and named hemslelis E.

### 2.2. Cytotoxic Activity

Compounds **1**–**5** were tested for their cytotoxic activities against three human tumor cell lines, Hela, HCT-8, and HepG-2, with the IC_50_ ranging from 5.9 to 33.9 μM compared to Cisplatin, the positive control (Table 2). It should be noted that none of the isolated compounds showed any selectivity in their cytotoxic activities. Compounds **2** and **3** displayed moderate activities towards HCT-8 human tumor cell lines, with the IC_50_ values of 5.9 and 6.1 μM, respectively. While, compound **4** was inactive against HCT-8, and compound **5** were inactive against both Hela and HepG-2. From the biological results, it can be noted that the oxidation of the fatty chain at C-17 in **5** may have decreased the activity. It was previously reported that some cucurbitane triterpenes that had similar structures to the compounds showed potent cytotoxic activities against several cancer cell lines

## 3. Discussion

Cucurbitane triterpenes, with the characteristics of a tetracyclic system and a fatty chain, were isolated from the tubers of *H. amabilis*, which are widely distributed in genus of *Hemsleya*. Ethnobotanically, the tubers of plant known as “xue dan” in the Yunnan province of China, and have been long used as a part of “Dai” medicine. Cucurbitane triterpenes have shown potent cytotoxic activity. As a result, we investigated all the isolated compounds for their cytotoxic activity. Compared with the cisplatin positive control group, all compounds displayed a measure of cytotoxic activities against human tumor cell lines, with the IC_50_ ranging from 5.9 to 33.9 μM. Further analysis of the data showed that compounds **2** and **3** displayed moderate activities towards HCT-8 human tumor cell lines over other compounds, while compound **5** was inactive against both Hela and HepG-2, which indicated that the oxidation of the fatty chain at C-17 in **5** may decreased the activity.

## 4. Materials and Methods

### 4.1. General Experimental Procedures

Optical rotation data were obtained using a Perkin-Elmer 341 digital polarimeter (Perkin-Elmer, Waltham, MA, USA). IR data were recorded using a Shimadzu FTIR-8400S spectrophotometer (Shimadzu, Kyoto, Japan). NMR data were obtained with a Bruker AV III 600 NMR spectrometer (Bruker, Billerica, MA, USA) (chemical shift values are presented as δ values with TMS as the internal standard) using the solvent Pyrridine-*d*_5_ as references. HRESIMS data were acquired using a LTQ-Obitrap XL spectrometer (Thermo Fisher Scientific, Waltham, MA, USA). HPLC was performed using a Lumiere K-1001 pump, a Lumiere K-2501 single λ absorbance detector, and an YMC-Pack ODS-A column (5 μm, 10 × 250 mm, YMC, Kyoto, Japan). Silica gel (100–200 mesh) and silica gel GF254 sheets (0.20–0.25 mm) (Qingdao Marine Chemical Plant, Qingdao, China) were used for column chromatography and TLC, respectively. TLC spots were visualized under UV light and by dipping into 5% H_2_SO_4_ in EtOH followed by heating. All solvents used were of analytical grade (Beijing Chemical Works). The cell lines were obtained from ATCC.

### 4.2. Plant Material

The tubers of *H. amabilis* were collected in Chongqing, Sichuan Province, People’s Republic of China, in August 2017, and were authenticated by Prof. Si-Rong Yi. A voucher specimen (CS170802) has been deposited at the Hebei University of Chinese Medicine.

### 4.3. Extraction and Isolation

The tubers of *H. amabilis* (1.2 kg) were powdered and extracted three times with 95% EtOH. The combined extract was concentrated under reduced pressure to furnish a dark brown residue (75.4 g), which was suspended in H_2_O and partitioned with petroleum ether, CH_2_Cl_2_, and EtOAc, respectively. The EtOAc fraction (13.3 g) was subjected to silica gel (100–200 mesh, 10 × 80 cm) column chromatography using a CH_2_Cl_2_-MeOH gradient (100:1; 80:1; 60:1; 40:1; 30:1; 20:1; 10:1; 5:1; 3:1) as eluent, and eight fractions were collected according to TLC analysis (Fr.Et1-Et8). Fr.Et2 (0.26 g) was purified by semi-HPLC with an isocratic of 78% MeOH-H_2_O on an YMC-Pack ODS-A column to get compounds **1** (5.7 mg) in *R*_t_ 17.4 min and **2** (6.3 mg) in *R*_t_ 20.3 min. Fr. Et3 (0.59 g) was separated by semi-preparative liquid chromatography using a MeOH-H_2_O (70:30) isocratic to yield **3** (7.4 mg, *R*_t_ 22.1 min) and **4** (6.7 mg, *R*_t_ 24.4 min). Compound **5** (7.9 mg, *R*_t_ 26.8 min) was obtained by semi-HPLC with a MeOH-H_2_O (64:37) system in Fr. Et5. The entire detection was under UV 210 nm and the flow rate was 2 mL/min.

### 4.4. Characterization of Compounds ***1–5***

hemslelis A (**1**), White amorphous solid (MeOH); ${\left[\alpha \right]}_{D}^{20}$ + 73.1 (*c* = 0.1, MeOH); IR (KBr) cm^−1^ 3447, 2938, 2854, 1687, 1011; ^1^H and ^13^C-NMR (Pyridine-*d*_5_): see (Table 1); HRESIMS *m*/*z* 539.2942 [M + Na]^+^ (calcd. 539.2985 for C_30_H_44_O_7_Na).

hemslelis B (**2**), White amorphous solid (MeOH); ${\left[\alpha \right]}_{D}^{20}$ + 35.7 (*c* = 0.1, MeOH); IR (KBr) cm^−1^ 3462, 2941, 2863, 1663, 1040; ^1^H- and ^13^C-NMR (Pyridine-d_5_): see (Table 1); HRESIMS *m*/*z* 509.3237 [M + Na]^+^ (calcd. 509.3243 for C_30_H_46_O_5_Na).

hemslelis C (**3**), White amorphous solid (MeOH); ${\left[\alpha \right]}_{D}^{20}$ + 44.8 (*c* = 0.1, MeOH); IR (KBr) cm^−1^ 3468, 2935, 2850, 1657, 1032; ^1^H- and ^13^C-NMR (Pyridine-d_5_): see (Table 1); HRESIMS *m*/*z* 511.3328 [M + Na]^+^ (calcd. 511.3399 for C_30_H_48_O_5_Na).

hemslelis D (**4**), White amorphous solid (MeOH); ${\left[\alpha \right]}_{D}^{20}$ + 58.4 (*c* = 0.1, MeOH); IR (KBr) cm^−1^ 3457, 2938, 2852, 1667, 1031; ^1^H- and ^13^C-NMR (Pyridine-*d*_5_): see (Table 1); HRESIMS *m*/*z* 525.3177 [M + Na]^+^ (calcd. 525.3192 for C_30_H_46_O_6_Na).

hemslelis E (**5**), White amorphous solid (MeOH); ${\left[\alpha \right]}_{D}^{20}$ + 79.4 (*c* = 0.1, MeOH); IR (KBr) cm^−1^ 3455, 2937, 2842, 1669, 1028; ^1^H- and ^13^C-NMR (Pyridine-*d*_5_): see (Table 1); HRESIMS *m*/*z* 639.3522 [M + Na]^+^ (calcd. 639.3509 for C_35_H_52_O_9_Na).

### 4.5. Acid Hydrolysis of ***5***

Compound **5** (2.0 mg) was heated in 3 mol/L CF_3_COOH (4 mL) for 3 h in a water bath. Each mixture was then extracted with EtOAc. The aqueous layer was evaporated to dryness with ethanol in vacuo at 50 °C until neutral. The residues were determined in comparison with d-Glucose using TLC (CHCl_3_:MeOH:H_2_O = 3:2:0.2, visualization with ethanol—5% H_2_SO_4_ spraying). Furthermore, the absolute configurations of the sugars were determined by gas chromatography according to a method previously described [17,18]. By this method, l-cysteine methyl ester hydrochloride (0.06 mol/L) and hexamethyldisilazane-trimethylchlorosilane (HMDS-TMCS, 3:1) were added to the aqueous residue for derivatization. The solution was then centrifuged and the precipitate removed. After these processes, n-hexane was used to extract derivate, which was then analyzed by GC. d-Glucose (t_R_ = 24.3 min) was detected by comparing with authentic monosaccharide.

### 4.6. Cytotoxic Bioassays

The cytotoxic activities of compounds **1–5** were evaluated using the MTT procedure with human cancer cell lines Hela (ATCC CCL-2), HCT-8 (ATCC CCL-244), and HepG-2 (ATCC HB-8065). The cells were incubated in DMEM (Dulbecco’s Modified Eagle Medium) supplemented with 10% fetal bovine serum and cultured at a density of 1.2 × 10^4^ cells/mL in a 96-well microtiter plate. Five different concentrations of each agent dissolved in dimethyl sulfoxide (DMSO) were then put in the wells. Each concentration was evaluated three times. After incubation under 5% CO_2_ at 37 °C for 48 h, 10 μL of MTT (4 mg/mL) was placed into each well, and the cells were incubated for an additional 4 h. Then, the liquid was taken out, and DMSO (200 μL) was put into the wells. The absorbance was documented with a microplate reader at a wavelength of 570 nm.

## Figures and Tables

**Table 1:** NMR data of compounds **1**–**5** (Pyridine-*d*_5_, 600 MHz).

No.	1		2		3		4		5	
	*δ*_C_	*δ*_H_ (*J* in Hz)	*δ*_C_	*δ*_H_ (*J* in Hz)	*δ*_C_	*δ*_H_ (*J* in Hz)	*δ*_C_	*δ*_H_ (*J* in Hz)	*δ*_C_	*δ*_H_ (*J* in Hz)
1	34.2	2.65, m; 1.73, m	21.7	2.28, m; 1.80, m	21.8	1.74, m; 2.00, m	21.7	2.29, m; 1.81, m	22.5	2.08, m; 1.63, m
2	70.5	4.14, td, 1.2, 8.4	29.6	1.87, m; 1.97, m	30.3	1.22, m; 1.95, m	29.6	2.10, m; 1.91, m	28.6	1.87, m; 2.40, m
3	80.8	3.52, d, 8.4	75.9	3.77, m	76.0	3.78, m	75.9	3.79, m	87.2	3.71, m
4	44.8		43.9		42.5		43.9		44.0	
5	168.3		169.2		145.1		169.3		168.4	
6	124.9	6.43, s	125.8	6.47, s	123.1	6.21, d, 9.0	125.8	6.47, s	125.4	6.31, s
7	200.3		199.6		66.5	4.42, dd, 9.0, 8.4	200.0		199.6	
8	58.8	2.81, s	60.1	2.69, s	54.4	2.50, d, 8.4	59.7	2.79, s	60.0	2.63, s
9	50.0		49.6		49.4		49.5		49.5	
10	36.6	3.25, m	38.1	3.09, m	36.5	2.64, m	38.1	3.08, m	38.0	2.99, m
11	211.5		212.0		214.6		212.4		211.6	
12	49.6	2.88, d, 15.0	49.1	3.01, d, 15.0 2.59, d, 15.0	41.5	2.57, d, 14.4 2.96, d, 14.4	50.2	2.95, d, 14.4 2.77, d, 14.4	49.1	2.91, d, 15.0 2.51, d, 15.0
		3.25, d, 15.0								
13	49.3		49.0		48.7		50.0		49.0	
14	48.8		49.6		49.2		49.6		49.7	
15	42.0	1.73, m; 2.76, m	35.3	1.38, m; 1.87, m	34.9	2.02, m; 1.53, m	35.2	2.08, m; 1.58, m	35.3	1.45, m; 1.88, m
16	70.9	5.17, m	28.5	1.23, m; 1.85, m	28.4	1.22, m; 1.81, m	22.4	1.41, m; 1.96, m	28.2	1.27, m; 1.85, m
17	56.2	3.70, d, 5.4	49.6	1.62, m	51.0	1.63, m	50.7	2.23, m	49.5	1.64, m
18	20.5	1.26, s	17.4	0.70, s	17.3	0.76, s	19.1	1.27, s	17.4	0.71, s
19	21.7	1.23, s	21.1	1.29, s	18.9	1.10, s	19.8	1.30, s	21.3	1.13, s
20	72.7		36.3	1.32, m	36.4	1.35, m	74.5		36.4	1.33, m
21	30.6	1.41, s	18.8	0.83, d, 6.6	18.9	0.85, d	27.8	1.46, s	18.9	0.80, d
22	46.9	2.02, m; 1.80, m	40.0	1.18, m; 1.52, m	35.0	1.21, m; 1.48, m	49.5	2.55, m; 2.49, m	40.0	1.81, m; 2.21, m
23	70.8	5.10, m	24.8	2.16, m; 2.30, m	24.9	2.18, m; 2.36, m	125.3	6.26, m	147.2	6.84, m
24	129.0	6.65, d, 6.6	127.7	5.90, t, 7.8	127.8	5.89, t, 7.2	139.9	6.06, d, 14.4	133.6	6.21, d, 15.6
25	139.3		141.4		141.3		73.7		198.2	
26	22.2	1.98, s	65.7	4.71, s	65.8	4.74, s	71.5	3.93, m		
27	61.2	4.50, d, 12.6	58.8	4.74, s	58.9	4.72, s	26.1	1.64, s	27.4	2.26, s
		4.57, d, 12.6								
28	22.0	1.50, s	19.0	1.10, s	22.1	1.78, s	28.4	1.22, s	18.9	1.06, s
29	23.4	1.36, s	28.4	1.21, s	27.8	1.18, s	19.0	1.20, s	28.6	1.20, s
30	25.1	1.45, s	26.1	1.44, s	26.7	1.47, s	26.0	1.45, s	25.6	1.57, s
Glu-1									107.6	4.85, d, 7.8
2									75.9	3.97, m
3									79.1	4.21, m
4									72.0	4.20, m
5									78.8	3.94, m
6									63.3	4.55, m; 4.39, m

**Table 2:** In vitro cytotoxic activities of compounds **1**–**5**.

Compounds	IC_50_ (μM)		
	Hela	HCT-8	HepG-2
**1**	12.5 ± 0.56 ^a^	14.7 ± 1.2	13.4 ± 0.54
**2**	26.8 ± 1.1	5.9 ± 0.85	24.2 ± 2.2
**3**	22.3 ± 0.89	6.1 ± 0.26	28.6 ± 1.1
**4**	31.5 ± 0.74	> 50	33.9 ± 2.0
**5**	> 50	18.2 ± 1.3	> 50
**Cisplatin** ^b^	0.78 ± 0.02	0.65 ± 0.05	0.18 ± 0.01

^a^ The values presented are the means ± SD of triplicate experiments. ^b^ Positive control substance.

## Supplementary Materials

The NMR spectra of compounds **1**–**5** (Figures S1–S30) are available online.
