# Supplementary material for: Novel Cucurbitane Triterpenes from the Tubers of Hemsleya amabilis with Their Cytotoxic Acitivity
Source: Molecules. 2019 Jan 17;24(2):331. doi: 10.3390/molecules24020331 (PMC6359121; doi:10.3390/molecules24020331)
Supplement: Supplementary file 1 [file molecules-24-00331-s001.pdf]

Novel cucurbitane triterpenes from the tubers of *Hemsleya amabilis* with their anti-tumor acitivity

Wei Feng [1,a], Yuan Zhou [1,a], Ling-Yu Zhou [1], Li-Ying Kang [1], Xiang Wang [1], Bao-Lin Li [1], Qing Li [1], and Li-Ying Niu [1,*]

[1] School of Pharmaceutical Sciences, Hebei TCM Formula Granule Technology Innovation Center& TCM Formula Granule Research Center of Hebei Province University, Hebei University of Chinese Medicine, Shijiazhuang 050091, China.

[a] These authors contributed equally to this work

* Correspondence: niuliyingyy@126.com (L. Niu); Tel.: +86 311 89926208 (L.Niu.)

Received: date; Accepted: date; Published: date

Abstract: The chemical research of the medicinal plant *Hemsleya amabilis* (Cucurbitaceae) yielded five new cucurbitane-type triterpenes hemslelis A-E (**1**-**5**) by silica gel column, ODS column, and semi-HPLC techniques. The structure was determined by spectroscopic analysis and examined alongside existing data from prior studies. Compound **1-5** was evaluated for their anti-tumor activity against three human tumor cell lines, Hela, HCT-8, and HepG-2, with the IC_{50} ranging from 5.9 to 33.9 μM compared to Cisplatin

Keywords: *Hemsleya amabilis*; cucurbitane-type; triterpenes; cytotoxic activity.

List of Figures S1-S30

Figure S1. ^{1}H-NMR (600 MHz, Pyridine-d_5) spectrum of the new compound **1**

Figure S2. ^{13}C-APT (150 MHz, Pyridine-d_5) spectrum of the new compound **1**

Figure S3. HSQC spectrum of the new compound **1**

Figure S4. HMBC spectrum of the new compound **1**

Figure S5. ^{1}H-^{1}H COSY spectrum of the new compound **1**

Figure S6. NOESY spectrum of the new compound **1**

Figure S7. ^{1}H-NMR (600 MHz, Pyridine-d_5) spectrum of the new compound **2**

Figure S8. ^{13}C-APT (150 MHz, Pyridine-d_5) spectrum of the new compound **2**

Figure S9. HSQC spectrum of the new compound **2**

Figure S10. HMBC spectrum of the new compound **2**

Figure S11. ^{1}H-^{1}H COSY spectrum of the new compound **2**

Figure S12. NOESY spectrum of the new compound **2**

Figure S13. ^{1}H-NMR (600 MHz, Pyridine-d_5) spectrum of the new compound **3**

Figure S14. ^{13}C-APT (150 MHz, Pyridine-d_5) spectrum of the new compound **3**

Figure S15. HSQC spectrum of the new compound **3**

Figure S16. HMBC spectrum of the new compound **3**

Figure S17. ^{1}H-^{1}H COSY spectrum of the new compound **3**

Figure S18. NOESY spectrum of the new compound **3**

Figure S19. ^{1}H-NMR (600 MHz, Pyridine-d_5) spectrum of the new compound **4**

Figure S20. ^{13}C-APT (150 MHz, Pyridine-d_5) spectrum of the new compound **4**

Figure S21. HSQC spectrum of the new compound **4**

Figure S22. HMBC spectrum of the new compound **4**

Figure S23. ^{1}H-^{1}H COSY spectrum of the new compound **4**

Figure S24. NOESY spectrum of the new compound **4**

Figure S25. ^{1}H-NMR (600 MHz, Pyridine-d_5) spectrum of the new compound **5**

Figure S26. ^{13}C-APT (150 MHz, Pyridine-d_5) spectrum of the new compound **5**

Figure S27. HSQC spectrum of the new compound **5**

Figure S28. HMBC spectrum of the new compound **5**

Figure S29. ^{1}H-^{1}H COSY spectrum of the new compound **5**

Figure S30. NOESY spectrum of the new compound **5**

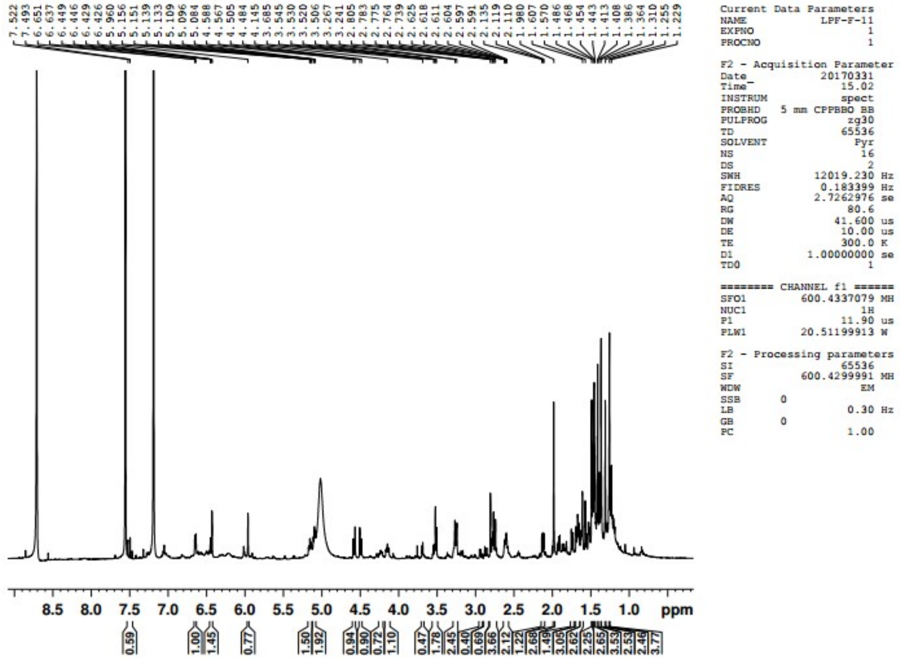

Figure S1. ^{1}H-NMR (600 MHz, Pyridine-d_5) spectrum of the new compound **1**

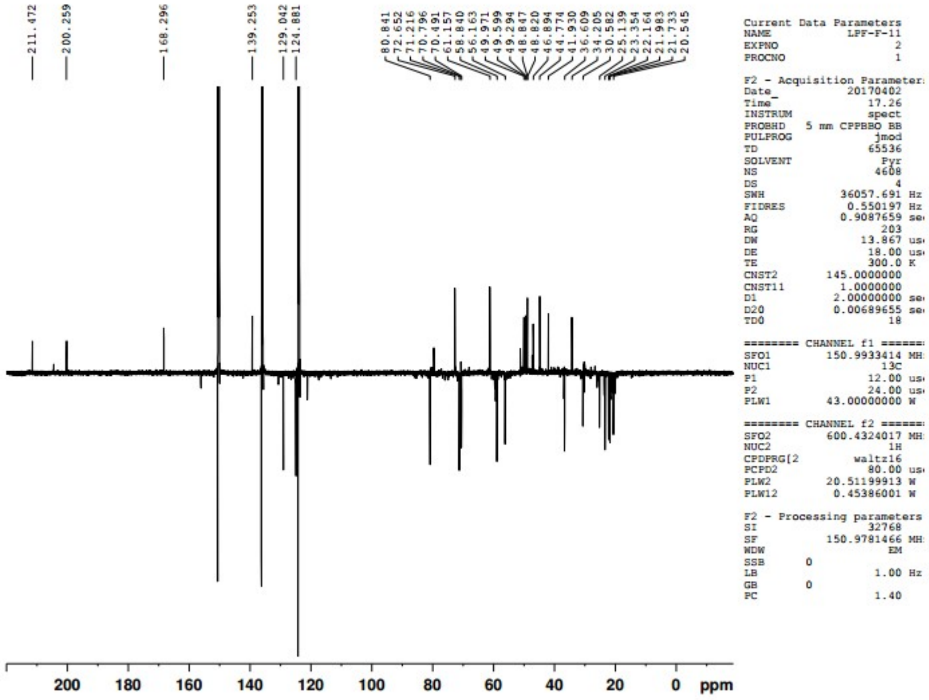

Figure S2. ^{13}C-APT (150 MHz, Pyridine-d_5) spectrum of the new compound **1**

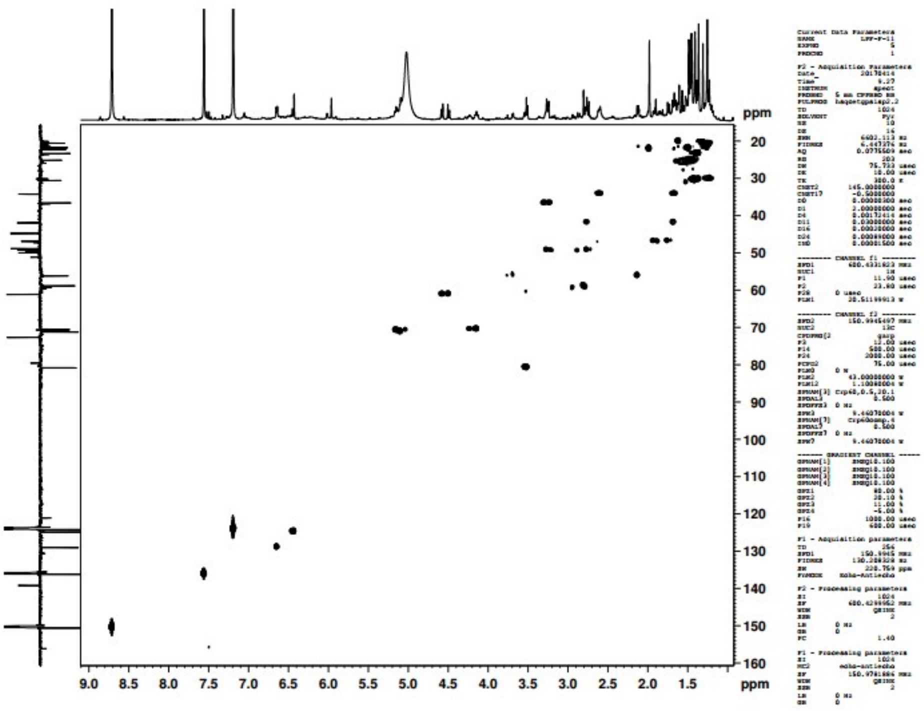

Figure S3. HSQC spectrum of the new compound **1**

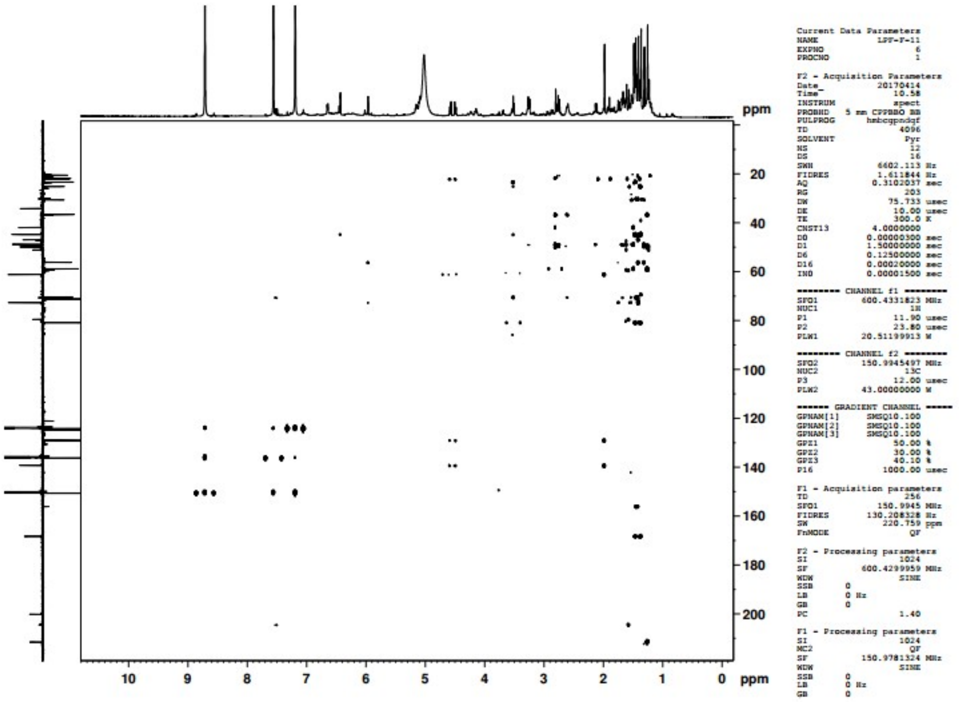

Figure S4. HMBC spectrum of the new compound **1**

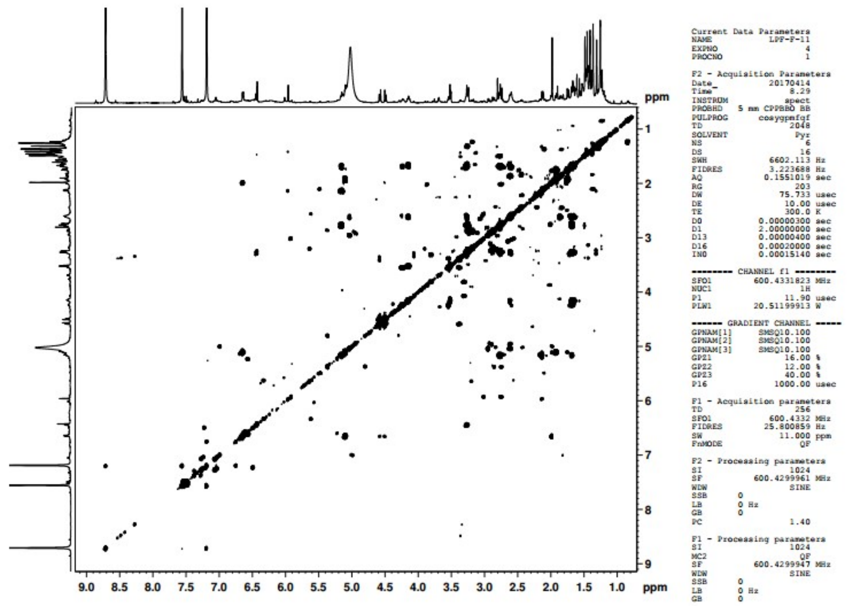

Figure S5. ^{1}H-^{1}H COSY spectrum of the new compound **1**

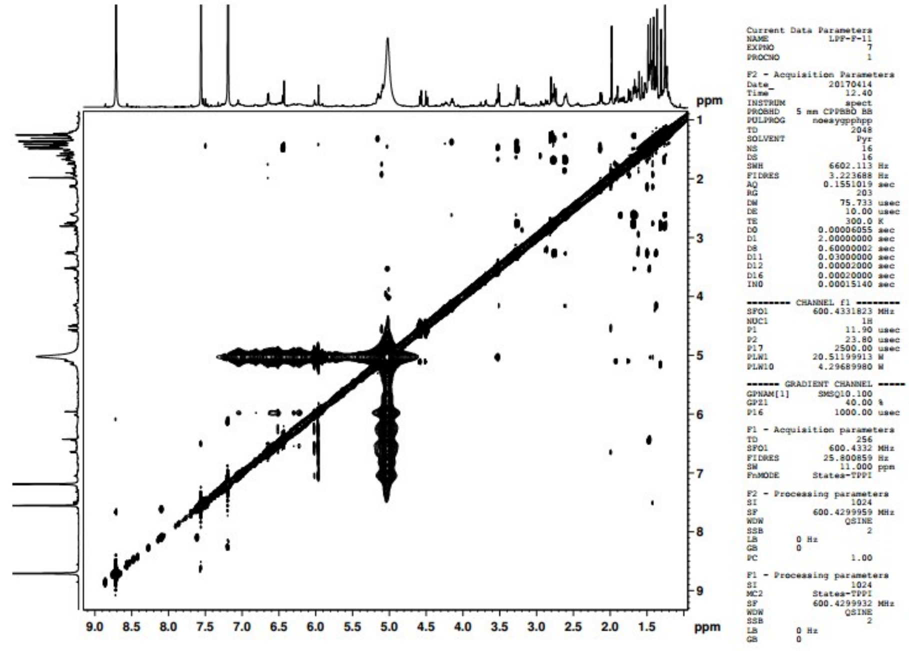

Figure S6. NOESY spectrum of the new compound **1**

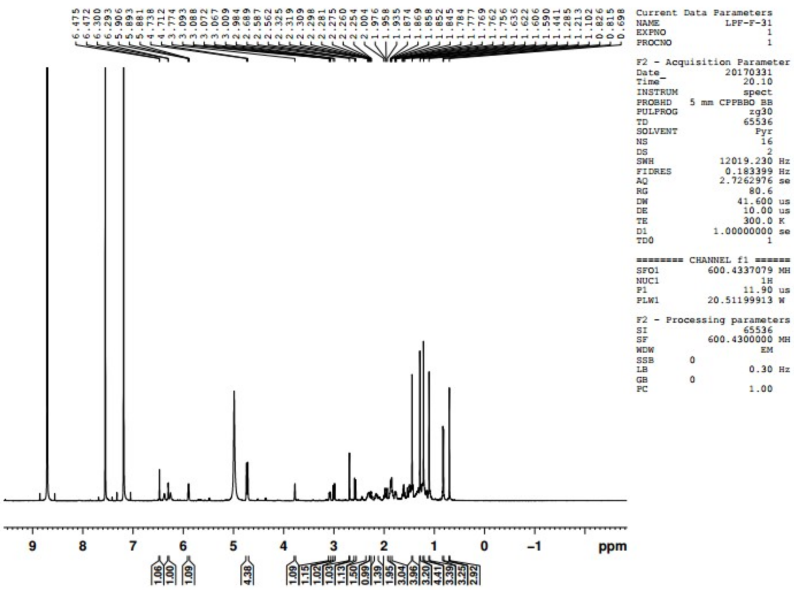

Figure S7. ^{1}H-NMR (600 MHz, Pyridine-d_5) spectrum of the new compound **2**

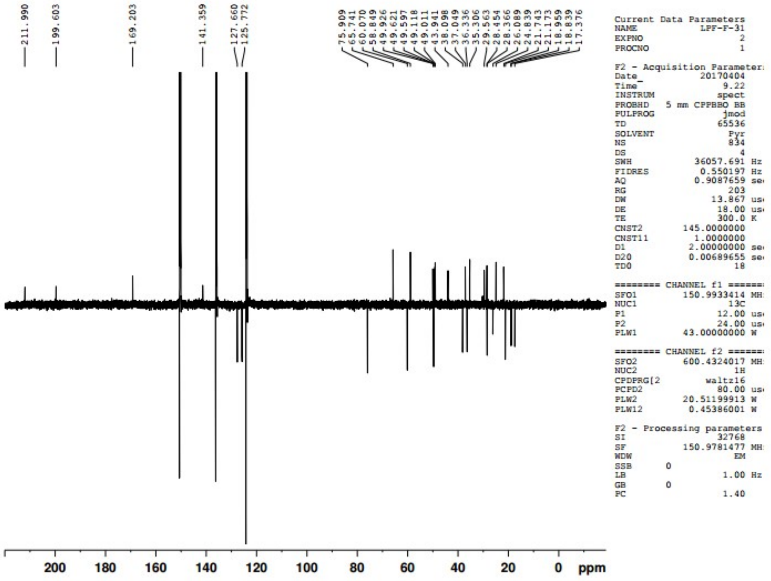

Figure S8. ^{13}C-APT (150 MHz, Pyridine-d_5) spectrum of the new compound **2**

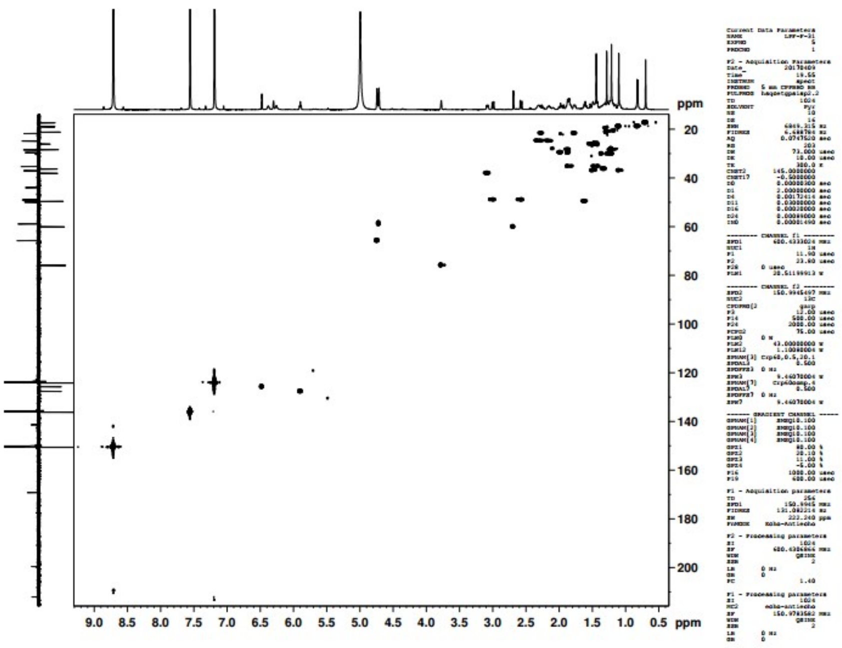

Figure S9. HSQC spectrum of the new compound **2**

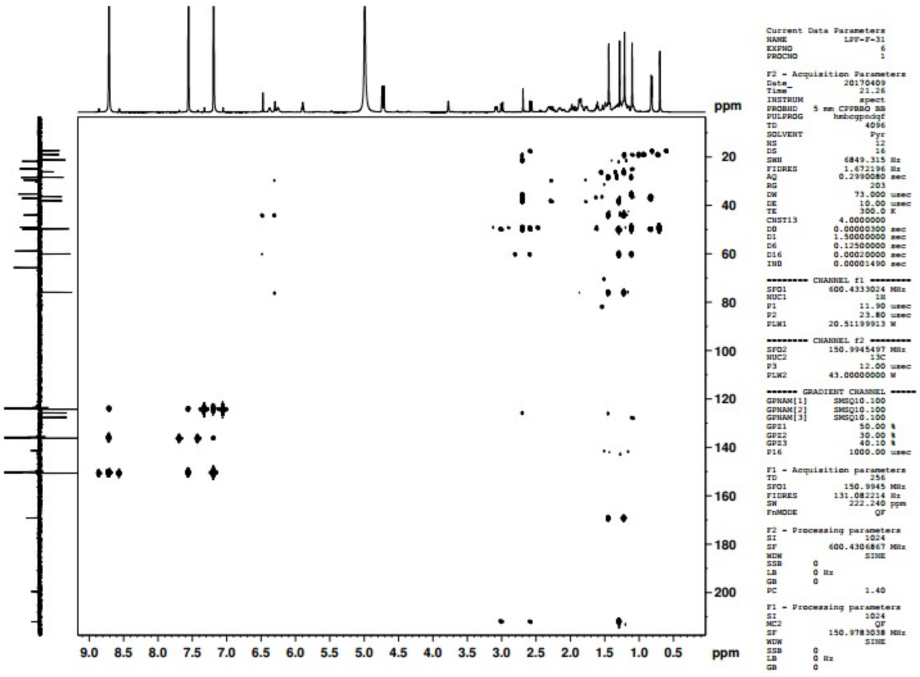

Figure S10. HMBC spectrum of the new compound **2**

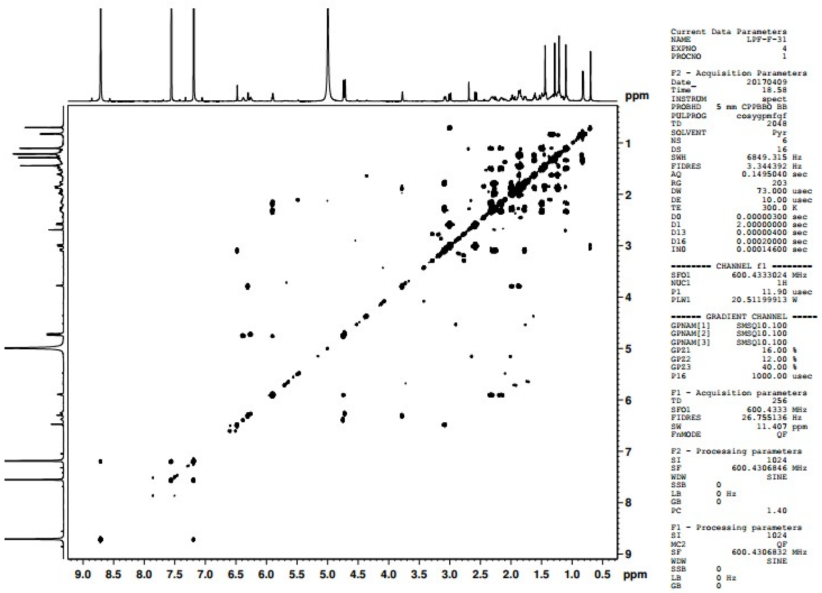

Figure S11. ^{1}H-^{1}H COSY spectrum of the new compound **2**

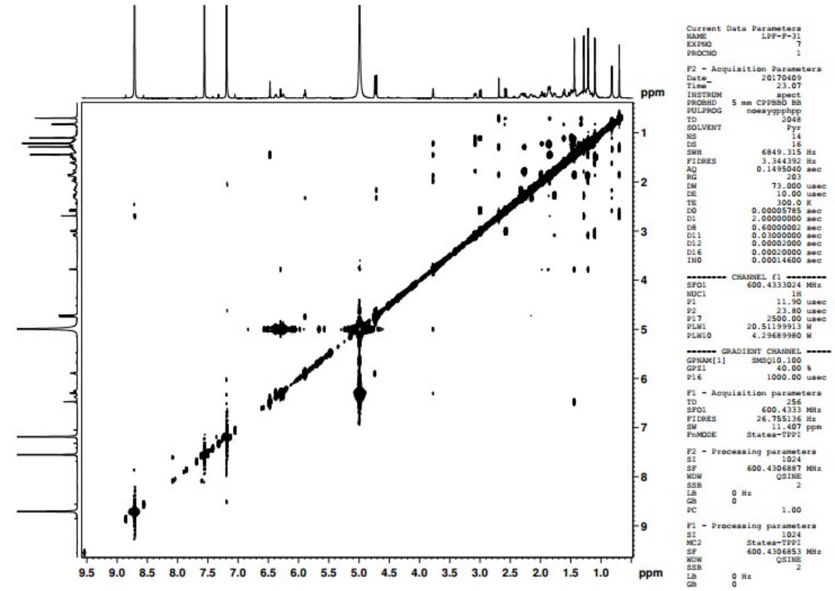

Figure S12. NOESY spectrum of the new compound **2**

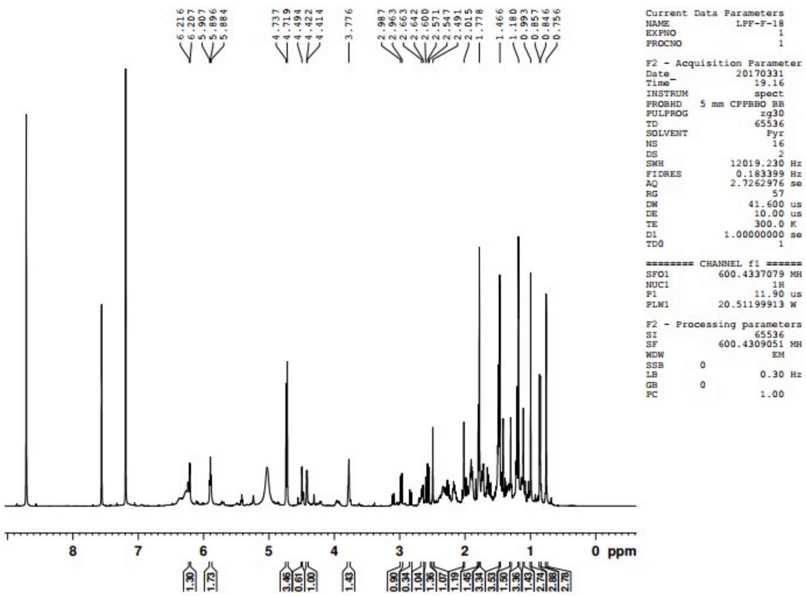

Figure S13. ^{1}H-NMR (600 MHz, Pyridine-d_5) spectrum of the new compound **3**

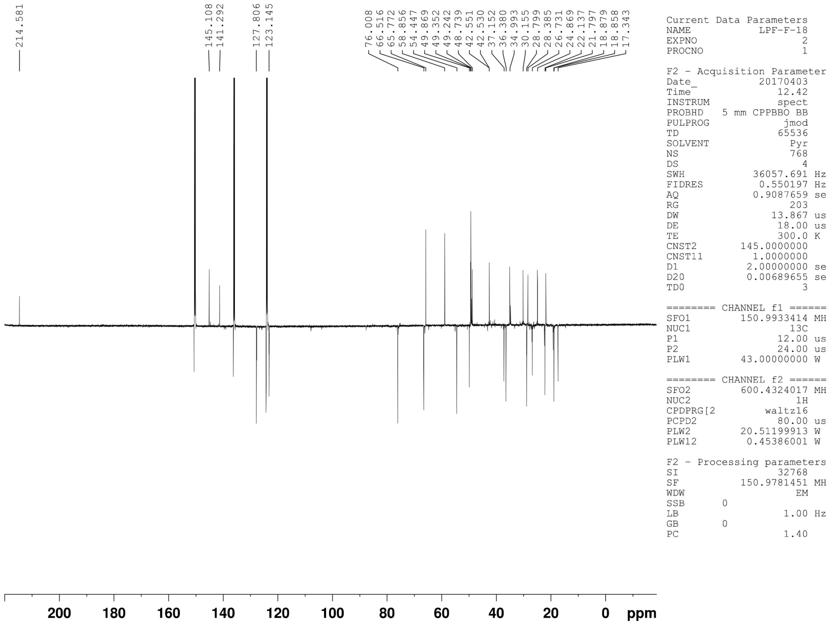

Figure S14. ^{13}C-APT (150 MHz, Pyridine-d_5) spectrum of the new compound **3**

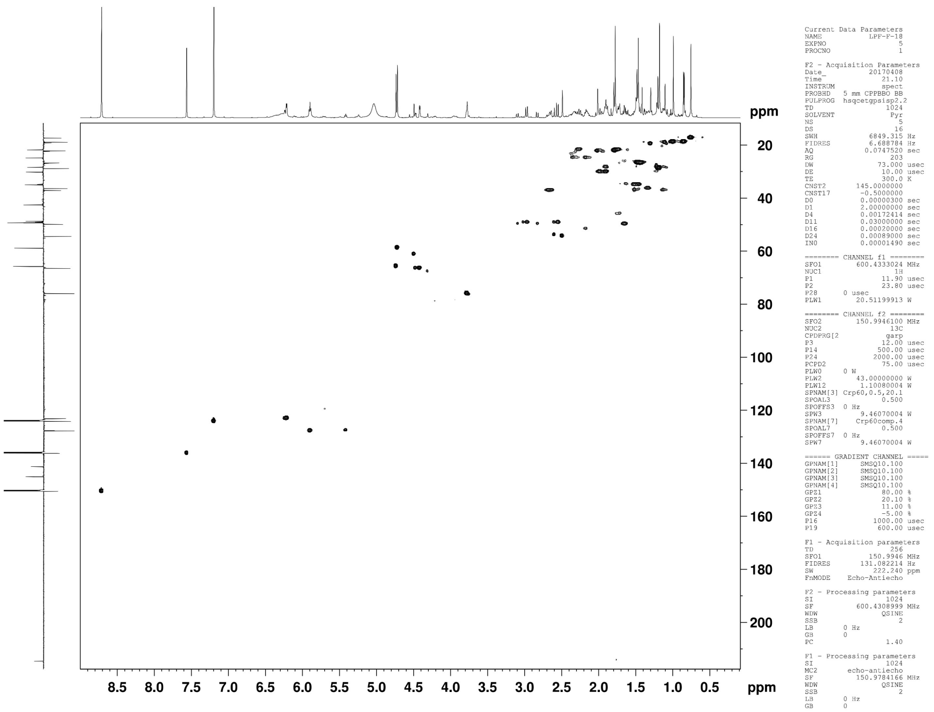

Figure S15. HSQC spectrum of the new compound **3**

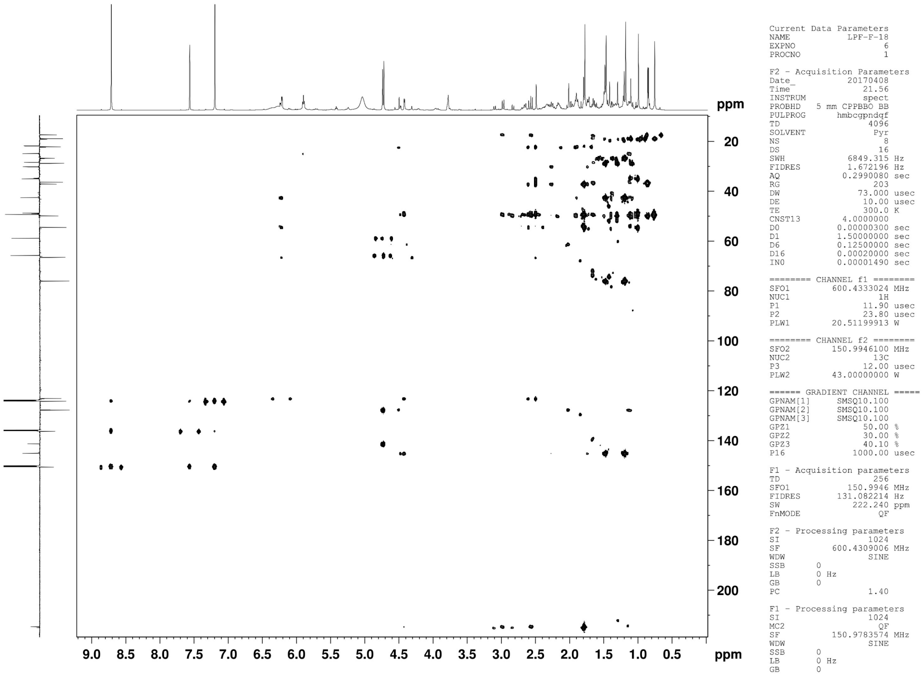

Figure S16. HMBC spectrum of the new compound **3**

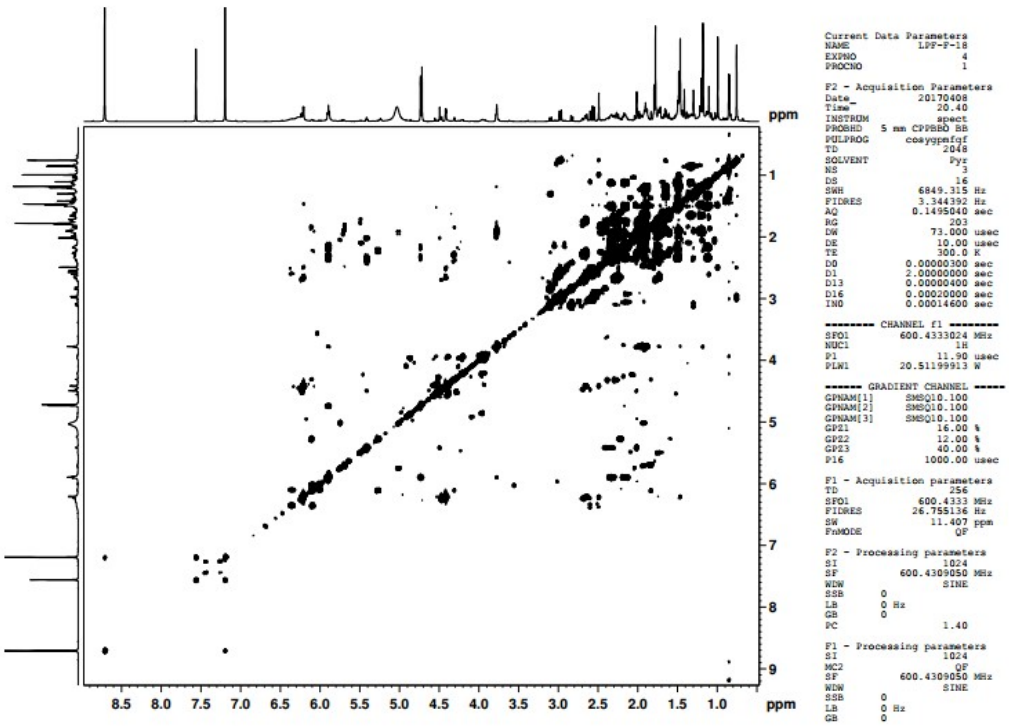

Figure S17. ^{1}H-^{1}H COSY spectrum of the new compound **3**

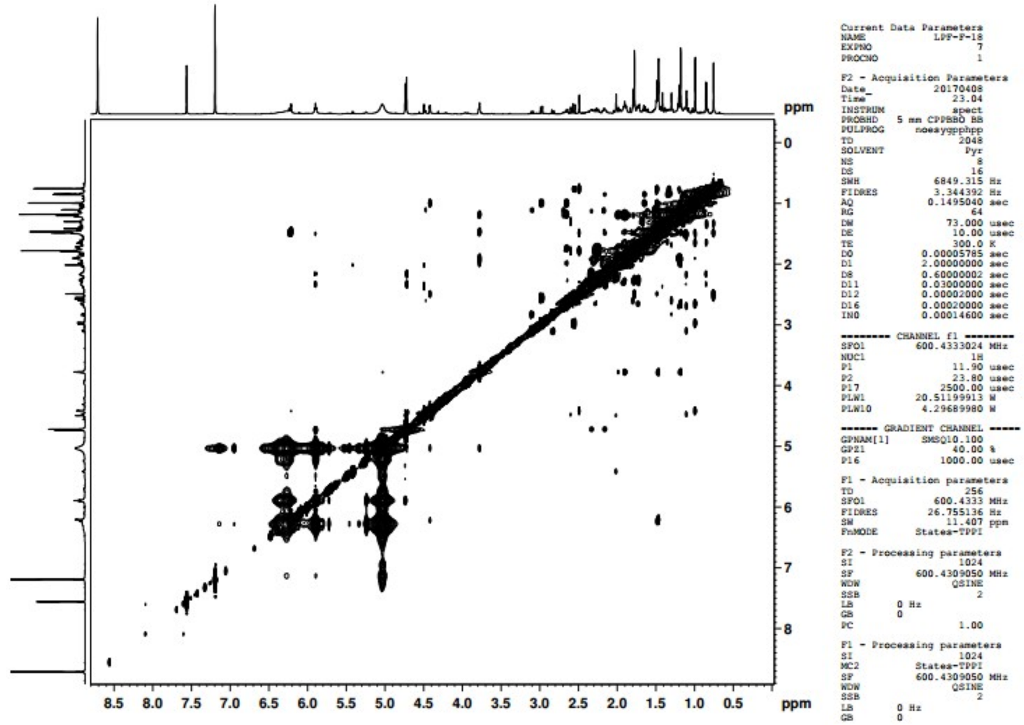

Figure S18. NOESY spectrum of the new compound **3**

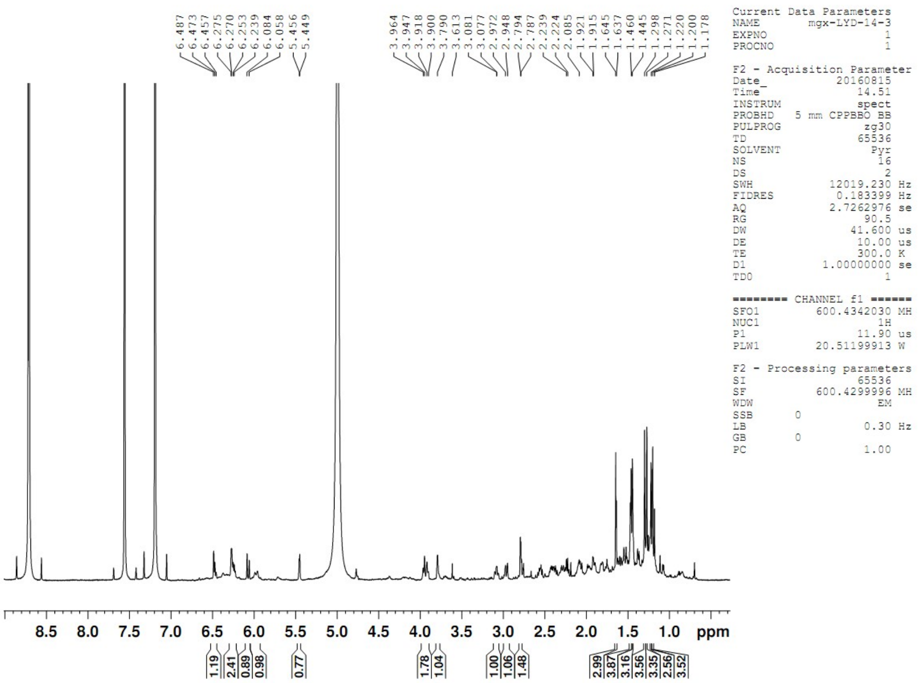

Figure S19. ^{1}H-NMR (600 MHz, Pyridine-d_5) spectrum of the new compound **4**

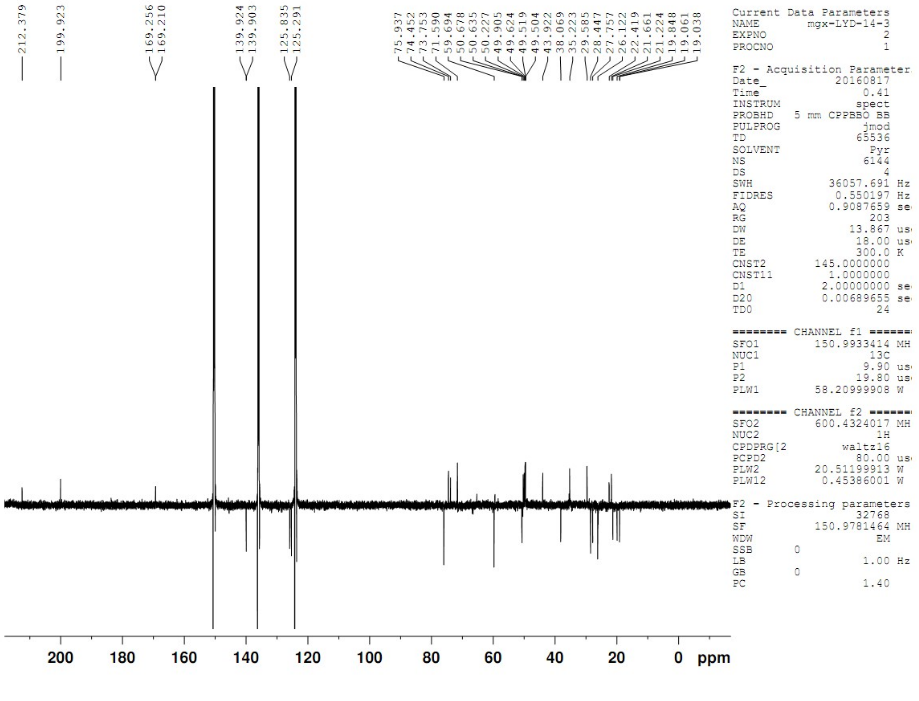

Figure S20. ^{13}C-APT (150 MHz, Pyridine-d_5) spectrum of the new compound **4**

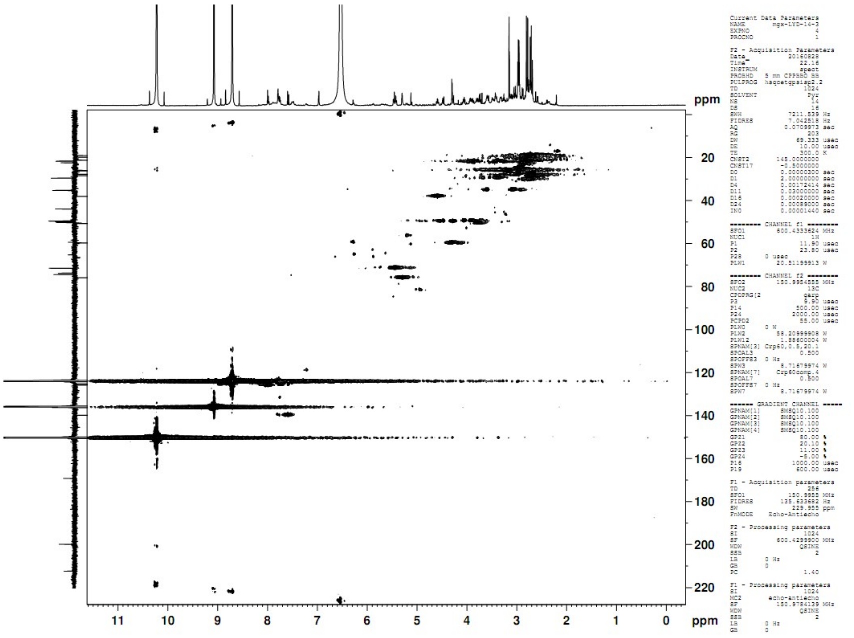

Figure S21. HSQC spectrum of the new compound **4**

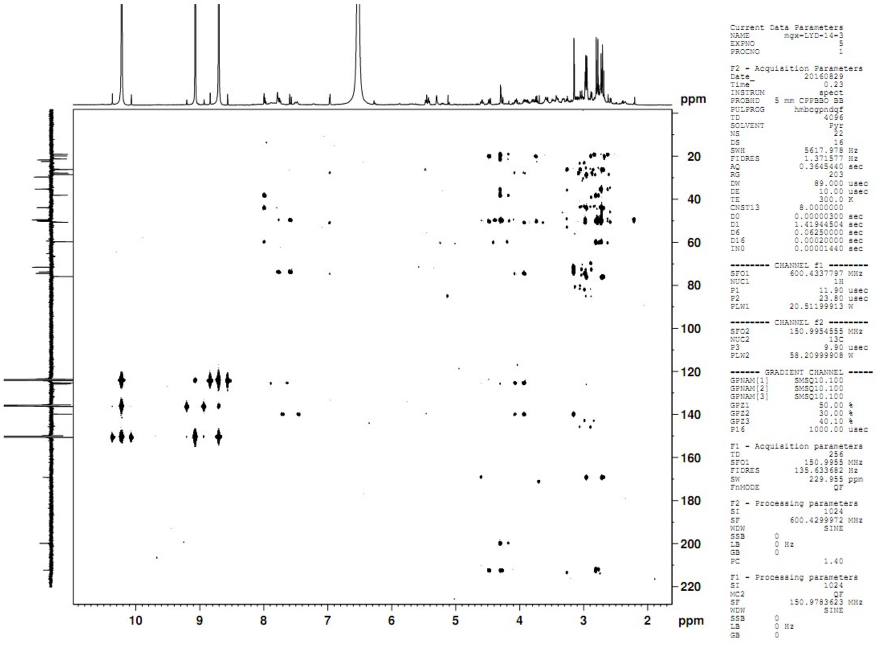

Figure S22. HMBC spectrum of the new compound **4**

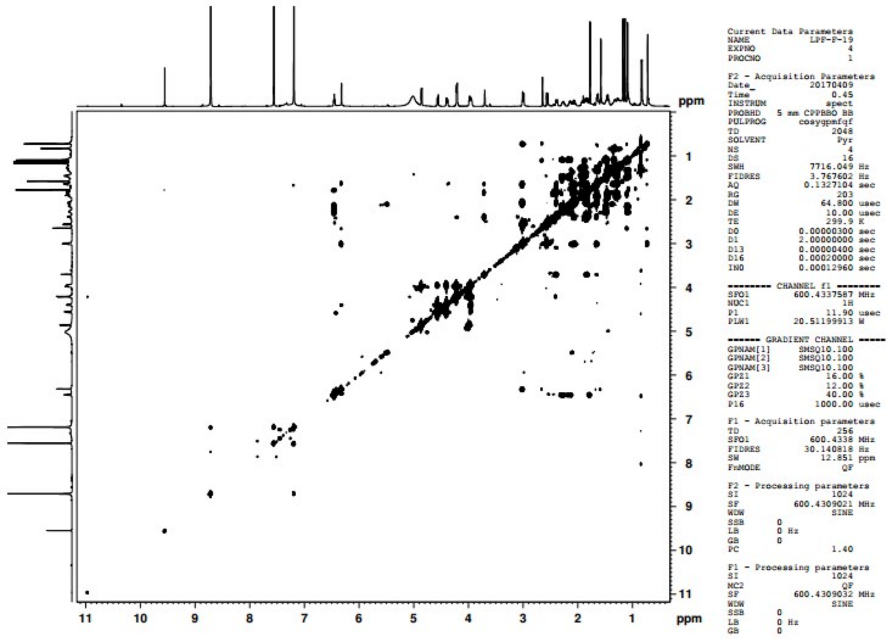

Figure S23. ^{1}H-^{1}H COSY spectrum of the new compound **4**

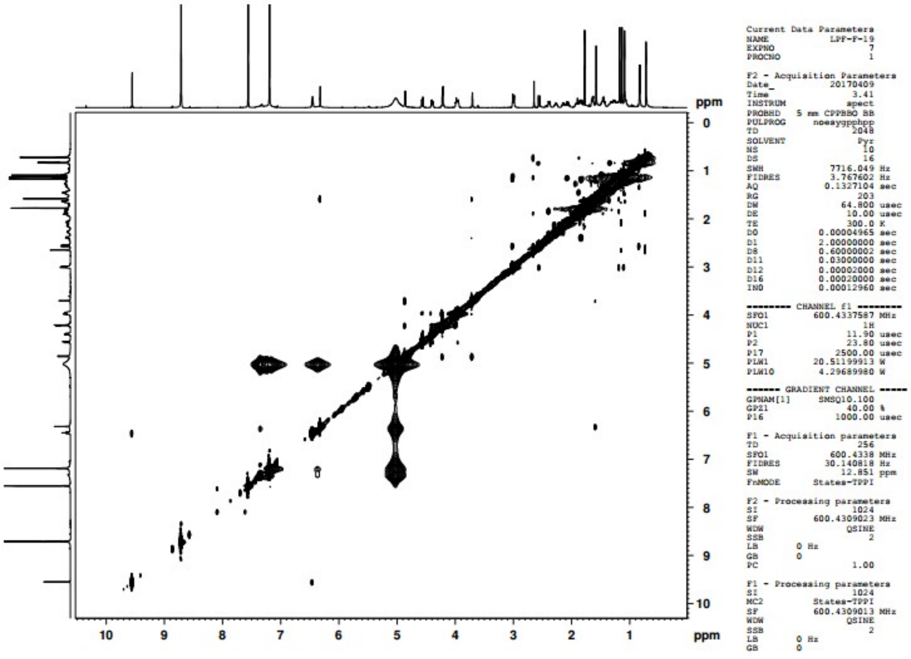

Figure S24. NOESY spectrum of the new compound **4**

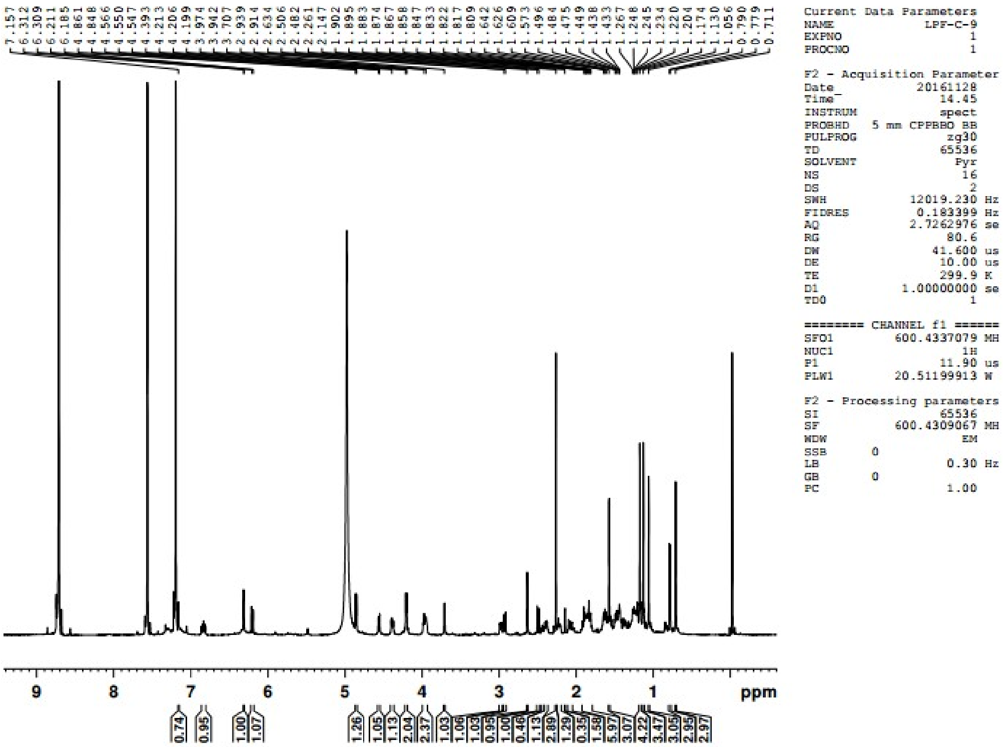

Figure S25. ^{1}H-NMR (600 MHz, Pyridine-d_5) spectrum of the new compound **5**

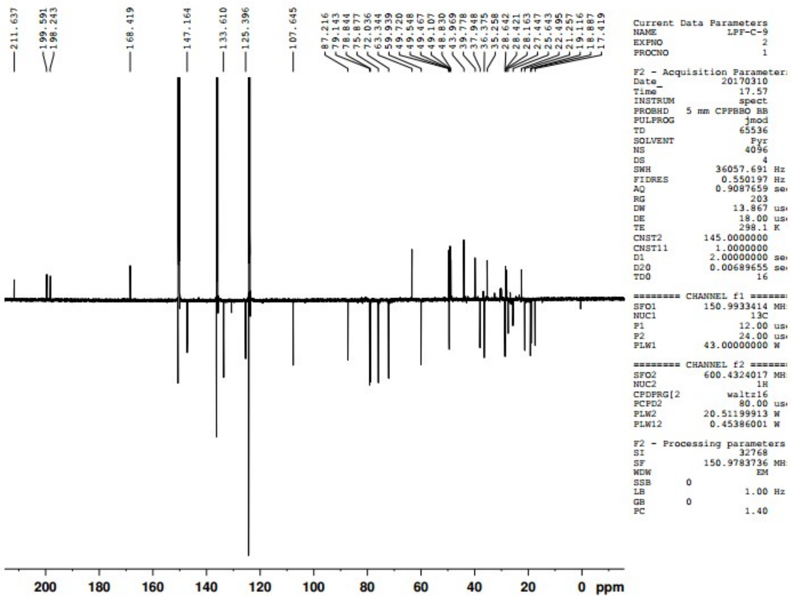

Figure S26. ^{13}C-APT (150 MHz, Pyridine-d_5) spectrum of the new compound **5**

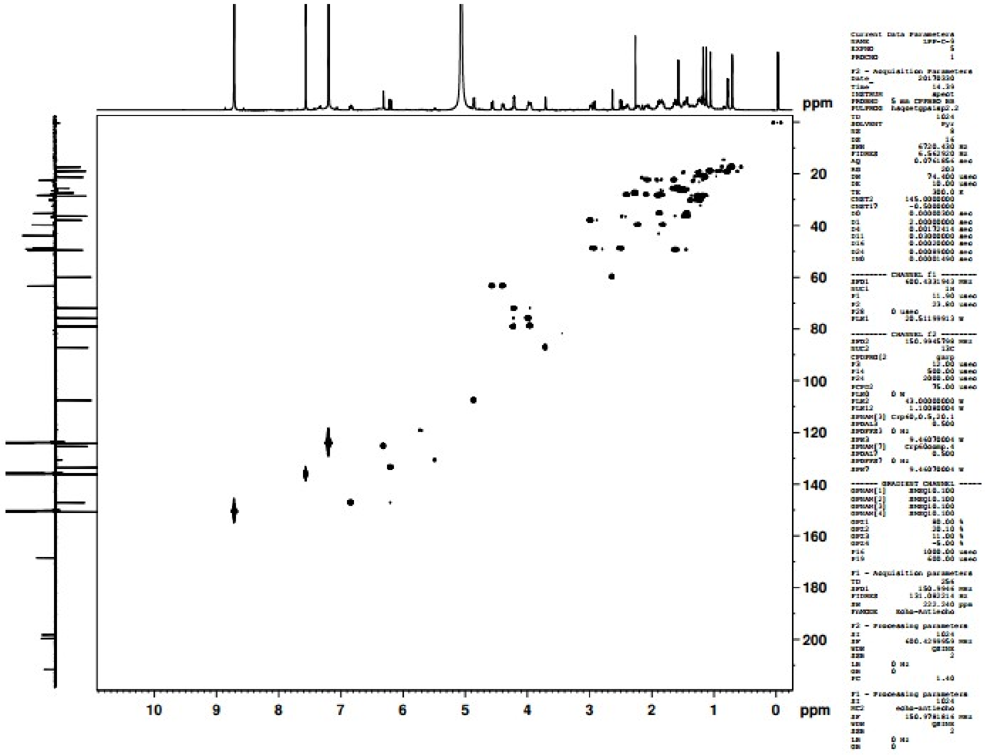

Figure S27. HSQC spectrum of the new compound **5**

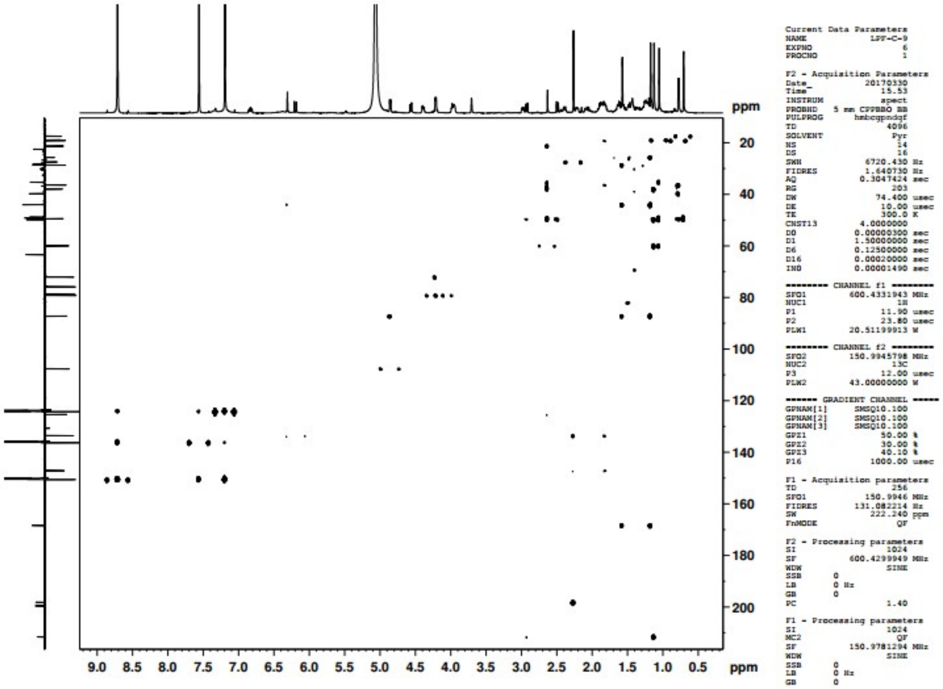

Figure S28. HMBC spectrum of the new compound **5**

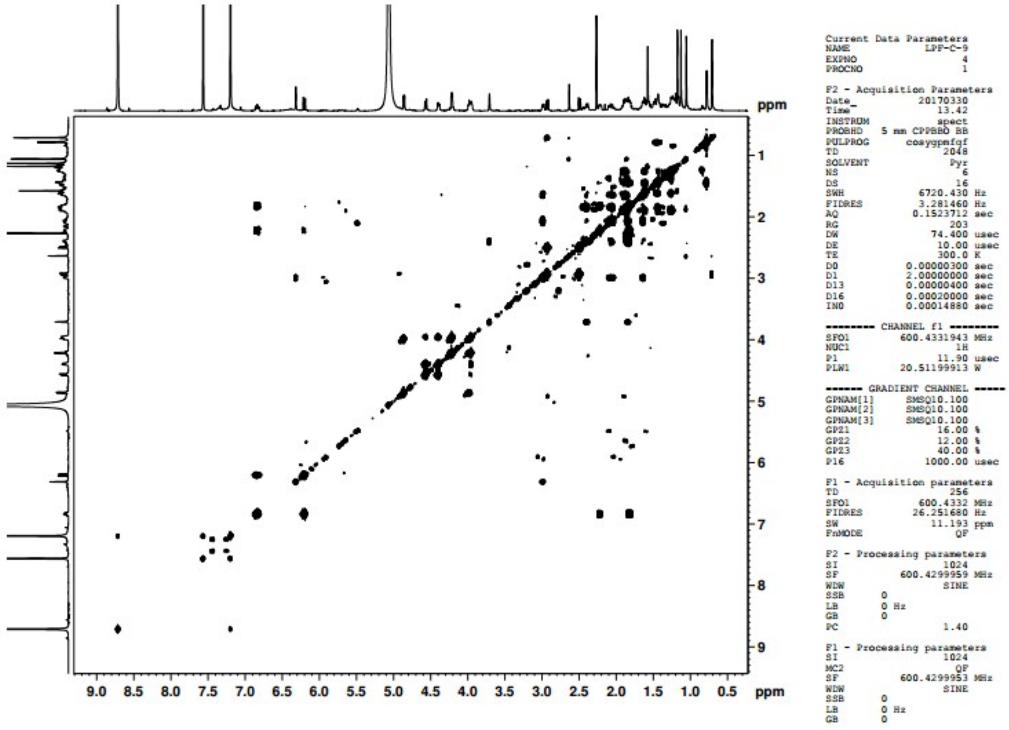

Figure S29. ^{1}H-^{1}H COSY spectrum of the new compound **5**

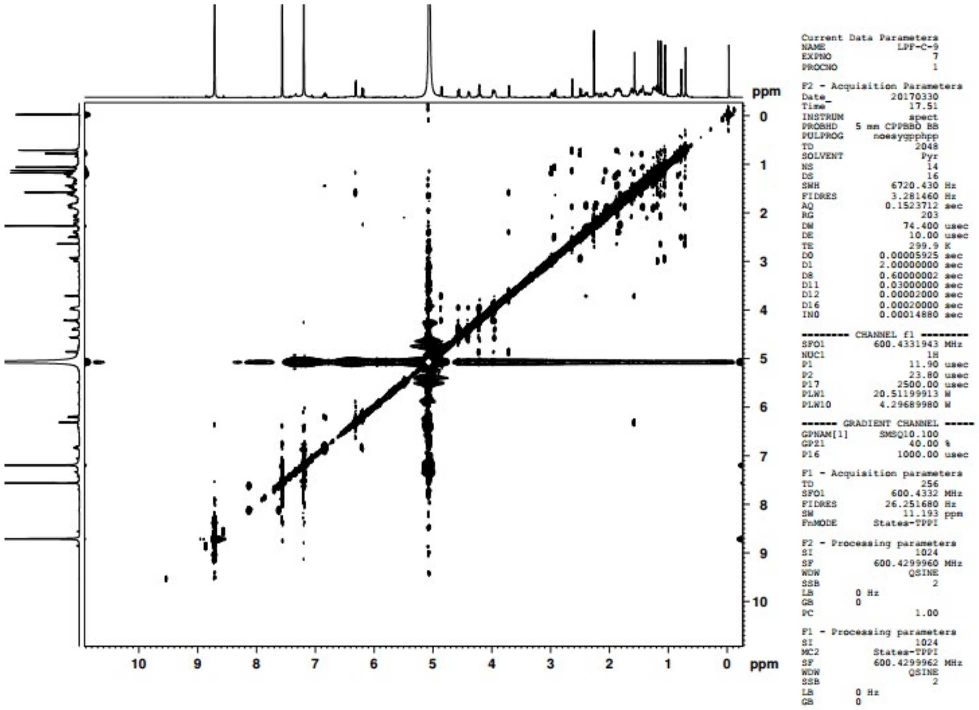

Figure S30. NOESY spectrum of the new compound **5**
